# Supplementary material for: Association of Circulating miR-145-5p and miR-let7c and Atherosclerotic Plaques in Hypertensive Patients
Source: Biomolecules. 2021 Dec 7;11(12):1840. doi: 10.3390/biom11121840 (PMC8699419; doi:10.3390/biom11121840)
Supplement: Supplementary file 1 [file biomolecules-11-01840-s001.zip › biomolecules-1490098-supplementary Figure S1.pdf]

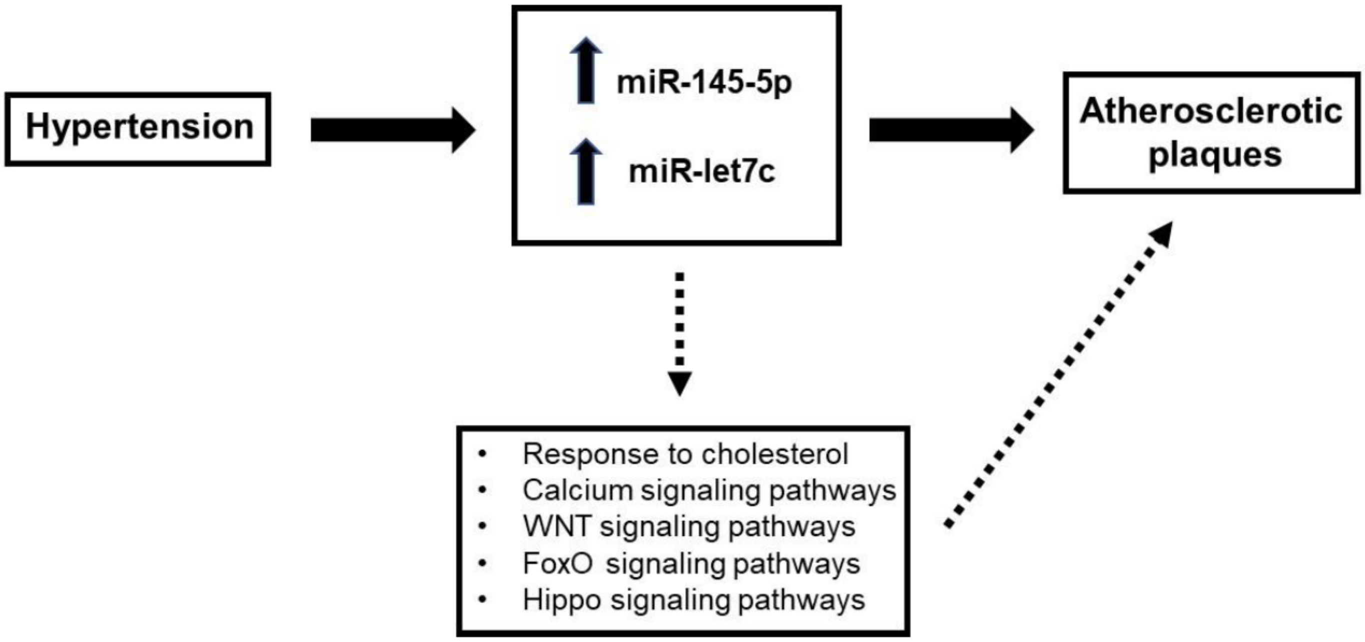

Figure S1. Summary of the findings.

Among hypertensive patients, elevated levels of mir-145-5p and Let-7c are associated with carotid atherosclerotic plaques. The dashed arrows indicate the potential mechanisms by which these miRNAs might modulate atherosclerosis based on gene ontology analysis.
